# Synergistic drug combination screening using a nanodroplet processing platform to enhance neuroblastoma treatment in TH‐MYCN transgenic mice

**DOI:** 10.1002/btm2.70007

**Published:** 2025-03-03

**Authors:** Yen‐Tzu Liao, Zhi‐Kai Yu, Yi‐Xun Huang, Kuan‐Hung Lin, Ching‐Te Kuo, Tsai‐Shan Yang, Pei‐Yi Wu, Chi‐Tai Yeh, Yen‐Lin Liu, Chien‐Chin Chen, Chiung‐Nien Chen, Wen‐Ming Hsu, Hsinyu Lee

**Affiliations:** ^1^ Department of Life Science National Taiwan University Taipei City Taiwan; ^2^ Department of Mechanical and Electro‐Mechanical Engineering National Sun Yat‐sen University Kaohsiung City Taiwan; ^3^ Division of Pediatric Surgery, Department of Surgery National Taiwan University Hospital Taipei City Taiwan; ^4^ Department of Life Science National Central University Taoyuan City Taiwan; ^5^ Department of Pediatrics, School of Medicine, College of Medicine Taipei Medical University Taipei City Taiwan; ^6^ Department of Pediatrics Taipei Medical University Hospital Taipei City Taiwan; ^7^ Department of Pathology Ditmanson Medical Foundation Chia‐Yi Christian Hospital Chiayi Taiwan; ^8^ Department of Surgery National Taiwan University Hospital Taipei City Taiwan

**Keywords:** BioNDP, chemotherapy, drug screening, neuroblastoma, synergy, TH‐MYCN mice

## Abstract

Neuroblastoma is a highly aggressive pediatric cancer with a poor prognosis, particularly in high‐risk (HR) cases characterized by MYCN amplification. The severe side effects associated with high‐dose chemotherapy further complicate treatment. Despite significant advancements in drug screening, traditional platforms remain limited due to their requirement for large cell quantities and their low translational success from bench to clinic. These limitations hinder the application of personalized medicine screening for patients with neuroblastoma. To address these challenges, we developed a Bioinspired Nanodroplet Processing (BioNDP) platform. This innovative platform allows for the simultaneous screening of multiple drug combinations while reducing the required number of cells to just 100 and minimizing assay volumes to 200 nL per well. Using BioNDP, we screened chemotherapeutic combinations of cyclophosphamide, doxorubicin, and vincristine in both the SK‐N‐DZ neuroblastoma cell line and primary neuroblastoma cells derived from TH‐MYCN transgenic mice. Our findings revealed a specific drug combination that exhibited significant synergistic cytotoxicity in neuroblastoma cells. This combination completely eradicated tumors and significantly improved survival rates in TH‐MYCN mice, without notable side effects. This study highlights the potential of the BioNDP platform in bridging in vitro and in vivo results, offering a promising strategy for personalized medicine in the treatment of HR neuroblastoma, with reduced toxicity and enhanced therapeutic efficacy.

**Translational Impact Statements**

This study leverages a nanodroplet processing platform to advance treatment strategies for high‐risk neuroblastoma. Using primary neuroblastoma cells derived from a transgenic mouse model, the platform identified a low‐dose, synergistic drug combination that eradicates tumors while minimizing toxicity. By overcoming the limitations of traditional screening methods, this innovative approach bridges laboratory and clinical research, offering a path toward safer and more effective personalized therapies for aggressive pediatric cancers.

## 1 INTRODUCTION

Drug screening is a crucial step in identifying potential therapeutic compounds. Despite decades of advancements in drug screening platforms, conventional methods still encounter significant limitations. A major challenge in translational medicine is the low success rate in translating findings from bench to clinical application,^1^, ^2^, ^3^ often due to the complexity of the tumor microenvironment, disparities between preclinical models and human physiology, and intra‐patient heterogeneity.^4^, ^5^, ^6^ These challenges underscore the need for innovative approaches. Personalized drug screening (PDS), which often requires patient‐derived samples, is increasingly recognized as a key advancement to address traditional method limitations. However, conventional cell‐based assays, which require large quantities of cells, restrict the scope of drug screenings when working with limited patient‐derived samples. To address this issue, we developed the Bioinspired Nanodroplet Processing (BioNDP) platform. This novel platform significantly reduces the required number of cells and enables simultaneous screening of multiple drugs.^7^, ^8^, ^9^, ^10^ The BioNDP platform, which integrates an array chip with an automatic liquid dispenser, facilitates drug combination screening using only 100 cells and a reaction volume of 200 nL per well. It allows for the evaluation of multiple drug combinations and has demonstrated effective drug screening for prostate and breast cancers, with efficacy validated in both xenograft mouse models and zebrafish models.^7^, ^11^ This innovative platform not only reduces cell consumption but also enhances the capability for extensive drug combination screening.

Neuroblastoma, a malignancy derived from neural crest cells, accounts for 8%–10% of childhood cancers and is responsible for over 15% of pediatric cancer‐related deaths.^12^, ^13^, ^14^ Patients with neuroblastoma are classified into low‐risk, intermediate‐risk, or high‐risk (HR) categories based on factors such as tumor stage defined by the International Neuroblastoma Risk Group Staging System, age at diagnosis, and genetic abnormalities.^15^, ^16^ MYCN amplification is particularly associated with HR neuroblastoma. The current therapeutic approach for HR neuroblastoma involves the aggressive administration of high‐dose chemotherapeutics, including cyclophosphamide (CP) + doxorubicin (DOX) + vincristine (VCR) (the combination is abbreviated CDV) and cisplatin + etoposide.^15^, ^17^, ^18^, ^19^ However, this treatment strategy often results in severe side effects, and responses can vary significantly among patients. These challenges underscore the urgent need for personalized and more effective combination chemotherapeutic strategies for neuroblastoma.^20^

The TH‐MYCN mouse model is widely used to study the mechanisms and therapeutic approaches for neuroblastoma.^21^ This genetically engineered model overexpresses the human MYCN oncogene under the control of the tyrosine hydroxylase (TH) promoter to specifically express in neural crest cells.^22^, ^23^, ^24^ This genetic modification closely mimics the MYCN amplification observed in human neuroblastoma, leading to spontaneous tumor development.^25^, ^26^ Furthermore, TH‐MYCN mice possess an intact immune system, allowing for comprehensive observations of drug responses and immune interactions.^27^, ^28^ The TH‐MYCN model accurately replicates both the genetic and pathological features of human neuroblastoma while preserving a functional immune system. Consequently, this animal model serves as a precise and reliable platform for preclinical testing in neuroblastoma research.^29^, ^30^

In this study, we sought to establish a PDS framework for neuroblastoma using the BioNDP platform. Our primary objective was to identify a drug combination that achieves superior therapeutic outcomes with reduced dosages compared to conventional treatments while minimizing adverse effects. To evaluate this approach, we employed TH‐MYCN mice as a model for neuroblastoma. Primary neuroblastoma cells were isolated from tumors in TH‐MYCN mice and exposed to various combinations of CP, DOX, and VCR—three standard chemotherapeutic agents used for HR patients with neuroblastoma. The BioNDP platform was used to determine the optimal drug combination, which was then validated for efficacy in TH‐MYCN mice. This study effectively bridges the gap between in vitro and in vivo research, establishing a robust preclinical model for developing personalized therapeutic strategies in neuroblastoma.

## 2 MATERIALS AND METHODS

### 2.1 Cell culture

The human neuroblastoma cell line SK‐N‐DZ (ATCC) and the human embryonic kidney cell line HEK‐293 (ATCC) were maintained in Dulbecco's Modified Eagle Medium (DMEM, HyClone), supplemented with 10% fetal bovine serum (FBS, Gibco) and 1% penicillin–streptomycin (GeneTeks Bioscience). Cells were cultured in a humidified incubator with 5% CO_2_ at 37°C. Isolated primary neuroblastoma cells were maintained in a specialized culture medium comprising 50% neurobasal medium (Gibco), 40% DMEM/F12 (Gibco), 10% FBS, 1X B‐27 minus vitamin A (Gibco), 1X N‐2 supplement (Gibco), 0.01 μg/mL epidermal growth factor (Gold Biotechnology), 0.02 μg/mL basic fibroblast growth factor (Gold Biotechnology), and 25 μg/mL primocin (InvivoGen).

### 2.2 The 384‐well plate cell viability assay

The diluted drug solutions were dispensed into the wells using the HP D300 digital dispenser, followed by dehydration under sterile conditions in a biosafety cabinet. Subsequently, 2000 SK‐N‐DZ cells were seeded into each well containing 25 μL of assay media. The cells were incubated for 24 h in a humidified incubator at 37°C with 5% CO_2_. Following incubation, ATP levels in each well were measured using the CellTiter‐Glo® luminescent cell viability assay (Promega), adhering to the manufacturer's protocol. Luminescence, indicative of cell viability, was quantified using the PHERAstar Detection System (BMG Labtech).

### 2.3 Drug combination screening on the BioNDP platform

The entire procedure is illustrated in Figure S1A and follows the methodology outlined in our previous study.^7^ The chip was sterilized using ultraviolet radiation for 30 min before use. Drug droplets of varying concentrations were then dispensed into the corresponding wells using a customized liquid handling dispenser (Versa 10 spotter, Aurora Instruments Ltd., Vancouver, CA). The dispensing operation exhibited remarkable stability, with a coefficient of variation (CV) as low as 0.8% (Figure S1B). Each droplet was precisely calibrated to a volume of 200 nL. After drug dispensing, the chip was incubated at room temperature for 10 min to evaporate the droplets, thereby converting the drug into powder form. A 200 nL droplet containing 100 cells was then dispensed into each well of the chip, which was subsequently sealed with a polydimethylsiloxane (PDMS)‐glass gasket. After 24 h of incubation, cell viability was assessed using the CellTiter‐Glo® luminescent cell viability assay, following the manufacturer's instructions (Promega). The luminescence intensities of live cells were captured using a UVP ChemStudio Plus (Analytik Jena) and analyzed with VisionWorks software (Analytik Jena).

### 2.4 Primary tumor cell isolation

Tumor samples were obtained from TH‐MYCN transgenic mice. Fresh neuroblastoma tumor samples were washed with phosphate‐buffered saline (PBS) containing 25 μg/mL primocin and then cut into 1–3 mm pieces. The tumor fragments were mixed with a primary neuroblastoma cell culture medium containing a blend of enzymes (Miltenyi Biotec) and transferred into a gentleMACS™ C Tube (Miltenyi Biotec). Tumor cell dissociation was carried out using a dissociator (RWD Life Science) per the equipment manual. Following dissociation, the cell suspension was filtered through a 70 μm strainer, and red blood cells were removed using red blood cell (RBC) lysis buffer (Gibco). Tumor cells were then purified through a two‐step isolation process. In the first step, non‐tumor cells were eliminated using a non‐tumor cell depletion kit (Miltenyi Biotec), following the manufacturer's instructions. In the second step, the cells were incubated with Biotin‐GD2 antibody (BioLegend) and isolated using a biotin targeting kit (Miltenyi Biotec), following the manufacturer's protocol to purify primary neuroblastoma cells expressing GD2.

### 2.5 Flow cytometry

Cells were collected and resuspended in 1× PBS containing 0.5% bovine serum albumin. The cells were then incubated with the fluorescein isothiocyanate (FITC)‐conjugated GD2 antibody (BioLegend, 357313). Flow cytometric analysis was conducted using a fluorescence‐activated cell sorting (FACS) Verse™ flow cytometer (BD Biosciences), and the data were analyzed with BD FACSuite software.

### 2.6 Animal experiment

This study adhered to the Guide for the Care and Use of Laboratory Animals of the National Institutes of Health. The 129/SvJ‐Tg(TH‐MycN) mouse strain^22^, ^31^ was kindly provided by Prof. Akira Nakagawara (MD, PhD, Chiba Cancer Center, Chiba, Japan). The breeding of female TH‐MYCN mice was conducted per the approved protocols of the Institutional Animal Care and Use Committee, College of Medicine, National Taiwan University. Hemizygous TH‐MYCN mice were provided by the Transgenic Mouse Core Facility at the College of Medicine, National Taiwan University. After weaning, hemizygous TH‐MYCN mice were monitored by ultrasound at the preaortic and adrenal areas to monitor spontaneous tumor growth. Sonograms were captured using the VisualSonics VEVO‐2100 High‐Frequency Ultrasound system (VisualSonics, Toronto, Canada). The screening strategy was optimized from Teitz et al.^12^ In this study, animals were anesthetized with 1.5% isoflurane in O₂ at a flow rate of 2 L/min. Hair removal cream (Nair) was applied to the abdominal and thoracic areas. For ultrasound scanning, animals were placed in a supine position on the imaging stage, which was coated with ultrasound transmission gel, and the ultrasound transducer (RMV‐706 at 40 MHz) was lowered stereotactically to the surface of the animal. Tumors, identified by irregular regions, were distinguished from normal tissue landmarks. During the imaging process, the maximal diameter of tumors was measured from axial and sagittal views, and tumor volume was calculated using the ellipsoid Equation (1)^32^, ^33^:

(1) $$\frac{1}{2}\times \text{width}\times \text{depth}\times \text{height}.$$

This approach has been widely used in the literature for tumor volume measurement in TH‐MYCN mouse models.^30^

Treatment was initiated, and treatment response was evaluated once to twice a week with ultrasound imaging once the maximal diameter of the tumor achieved 0.5 cm or larger. These mice were randomly assigned to receive intraperitoneal injections of the following treatments: vehicle (0.9% saline) for 3 days, 12 mg/kg CP (Baxter Healthcare Corporation) for 2 days, 0.6 mg/kg DOX (Pfizer) for 3 days, 0.06 mg/kg VCR (Pfizer) for 3 days, or a combination of these three drugs (Figure S2). The treatment schedule was based on a commonly used regimen in recent clinical trials and the National Comprehensive Cancer Network clinical practice guidelines for HR neuroblastoma.^15^, ^34^ The study was conducted over 12 weeks, with mice humanely euthanized if the maximal diameter of tumors reached 1.5 cm during this period.

The tumor response percentage for each mouse was calculated using the following Equation (2):

(2) $$\text{Tumor response}\%=\frac{b_{x}-a}{a}\times 100\%,$$

where *a* represents the tumor volume measured on the day when the tumor's maximum diameter exceeds 0.5 cm, *x* is the number of days post‐administration, and *b* is the tumor volume on day *x*.

The schedule for biochemical and hematological analyses in mice was aligned with the in vivo efficacy study described above. Whole blood and serum samples were collected from all mice 1 day after the final administration and analyzed by Bio‐Cando Incorporation (Taoyuan, Taiwan).

Tumors and organs, including the liver, spleen, and kidneys, were collected from mice on the day of sacrifice upon reaching the experimental endpoint. The organ samples were sent to the National Taiwan University Animal Resource Center for fixation, slicing, and hematoxylin and eosin (H&E) staining, and the histological examination was performed by Dr. Chen from Chia‐Yi Christian Hospital.

### 2.7 In vivo dosage calculation for animal experiments

The calculation process, which has been reported in our previous study, considers key factors including the Dose‐Reduction Index (DRI), maximum tolerated dose (MTD), and administration frequency (*n*). DRI values were calculated based on the single‐drug dose–response experiments of CP, DOX, and VCR using primary neuroblastoma cells on the BioNDP platform. The single‐drug response data were presented in Figure S4. Drug combination studies were subsequently performed on the same platform. The drug concentration‐effect data (cytotoxicity) were input into CompuSyn software,^35^, ^36^, ^37^ which calculated the DRI values for the three drugs: CP (3.16), DOX (6.74), and VCR (139.50). The averaged DRI across the combination was 49.8, reflecting the extent of dosage reduction enabled by the synergy of the drug combination. The MTD values of murine for CP, DOX, and VCR were obtained from the literature.^38^, ^39^, ^40^ Based on these studies, the following MTD values were used: CP: 300 mg/kg; DOX: 10 mg/kg; VCR: 1 mg/kg. The total number of drug administrations was based on the experimental design: CP: administered for 2 days over the treatment period; DOX: administered for 3 days over the treatment period; VCR: administered for 3 days over the treatment period.

The in vivo dosage for each drug was calculated using the following Equation (3):

(3) $$\text{In vivo dosage}=\left[\frac{\mathrm{MTD}}{\text{Averaged}\;\mathrm{DRI}}\right]\times n.$$

Using the formula, the dosages were determined as follows: CP: 300/49.8 × 2 = 12 mg/kg; DOX: 10/49.8 × 3 = 0.6 mg/kg; VCR: 1/49.8 × 3 = 0.06 mg/kg.

### 2.8 Statistical analysis

One‐way analysis of variance; This is a statistical tool for means comparison if different groups (ANOVA) was conducted to compare data across multiple groups and determine statistical significance. The half‐maximal inhibitory concentration (IC_50_) values were fitted and evaluated using a variable slope (four‐parameter) logistic equation in GraphPad Prism 9.0.0. The CV percentage of droplet dispensing on the chip was calculated based on luminescence intensity using the following Equation (4).

(4) $$\mathrm{CV}\%=\frac{\text{standard deviation}}{{\text{Signal}}_{\mathrm{ave}}}\times 100\%.$$

The percentage of live cells, derived from the detected luminescence intensity, was calculated using the following Equation (5):

(5) $$\text{Cell viability}\%=\frac{\gamma -\beta }{\alpha -\beta }\times 100\%.$$

where *α* represents the luminescence signal from wells without any treatment, *β* represents the luminescence signal from wells without cells (serving as the background signal), and *γ* represents the luminescence signal from wells with the selected treatments.

Drug combination synergy was assessed using Combination Index (CI) values calculated with CompuSyn^35^ software, classifying interactions as synergistic (CI <1), additive (0 ≤ CI ≤ 1), or antagonistic (CI >1).

## 3 RESULTS

### 3.1 Synergistic chemo‐drug combination screening in SK‐N‐DZ neuroblastoma cell line through BioNDP platform

**FIGURE 1:**
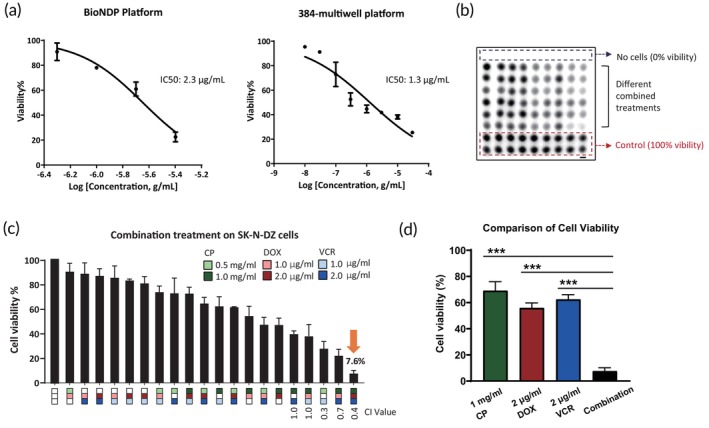
Identification of a synergistic chemo‐drug combination in the SK‐N‐DZ cell line using the Bioinspired Nanodroplet Processing (BioNDP) platform. (a) The dose–response assessment of doxorubicin (DOX) was analyzed in the SK‐N‐DZ cell line using a 384‐well plate via the BioNDP platform, with cell viability determined by the CellTiter‐Glo® luminescent cell viability assay. (b) Luminescence detection was performed on live SK‐N‐DZ cells following various combination treatments for 24 h. Cyclophosphamide (CP) (0.5 and 1 mg/mL), DOX (1 and 2 μg/mL), and vincristine (VCR) (1 and 2 μg/mL) were applied in this experiment. (c) Quantitative analysis of panel (b) is presented, with the refined drug combination showing a Combination Index (CI) value of 0.4, indicated by an orange arrow. (d) A comparison of cell viability between single‐drug treatments and the optimal combination selected from panel (c) is shown. Data are presented as mean ± standard deviation (SD from three independent experiments. A one‐way ANOVA was conducted to compare the cell viability of the combination treatment with each single‐drug treatment, with significant differences observed (***p < 0.001).

Previously, we successfully demonstrated the applicability of the BioNDP platform for drug screening in breast and prostate cancers.^7^ In this study, we expanded its application to neuroblastoma by evaluating the efficacy of DOX in the SK‐N‐DZ cell line, which is characterized by MYCN amplification. Cells were treated with DOX in 384‐well plates and on BioNDP chips, followed by a 24‐h incubation period. Subsequently, cell viability was assessed using the CellTiter‐Glo assay, which measures ATP levels as an indicator of metabolically active cells. The IC_50_ values were found to be comparable between the two platforms: 1.3 μg/mL for the 384‐well plate and 2.3 μg/mL for the BioNDP chip, underscoring the reliability of BioNDP in drug assessment (Figure 1a). To determine the appropriate concentrations of CP and VCR for subsequent combination screenings, we evaluated drug responses in SK‐N‐DZ cells using the BioNDP platform. The IC_50_ values for CP and VCR were determined to be 2.0 mg/mL and 2.1 μg/mL, respectively (Figure S3). Based on these efficacy tests, two concentrations of each drug were selected for screening potential synergistic combinations on the BioNDP platform. Notably, the combination of 1 mg/mL CP, 2 μg/mL DOX, and 2 μg/mL VCR resulted in a significant reduction in cell viability to 7.6%, indicating optimized cytotoxicity in SK‐N‐DZ cells (Figure 1b,c). In contrast, individual treatments exhibited lower cytotoxic effects (Figure 1d), highlighting the enhanced cytotoxic potential of the selected drug combination. To evaluate the synergistic effect of the drug combinations, we calculated the CI value, which quantitatively determines drug interactions.^41^ The CI value for the combination of 1 mg/mL CP, 2 μg/mL DOX, and 2 μg/mL VCR was 0.4 (Figure 1c), indicating a synergistic cytotoxic effect on SK‐N‐DZ cells. In summary, these initial results underscore the robustness and potential feasibility of the BioNDP platform in identifying synergistic drug combinations for neuroblastoma.

### 3.2 Identification for an optimal synergistic drug combination in primary neuroblastoma cells isolated from TH‐MYCN transgenic mice

**FIGURE 2:**
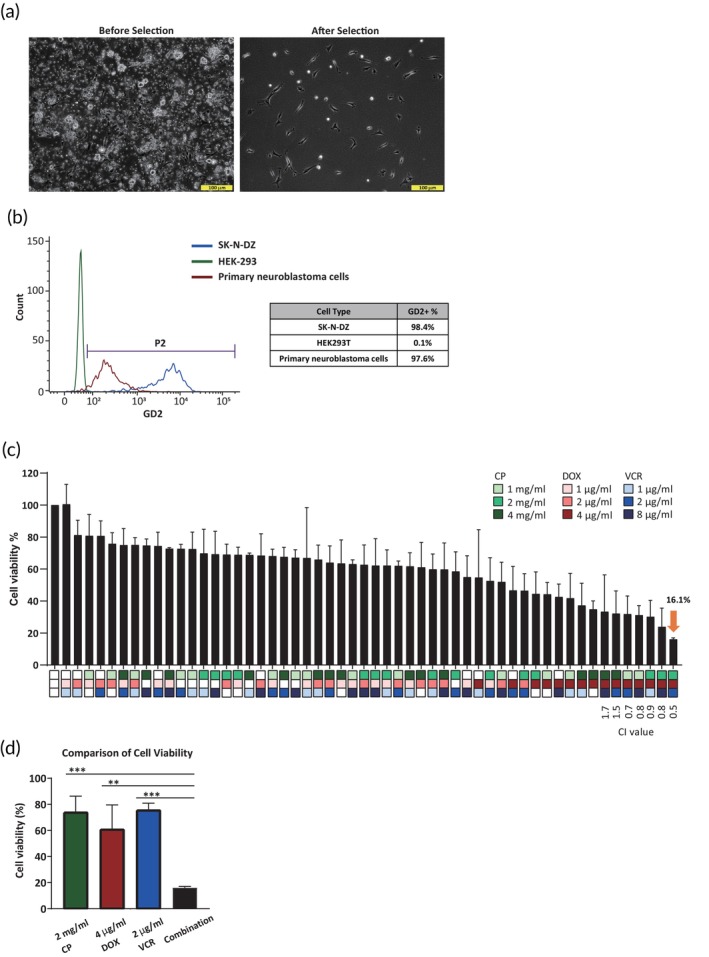
Screening for synergistic drug combinations in primary neuroblastoma cells derived from TH‐MYCN mice. (a) Brightfield images display the morphology of cells before (left) and after (right) the optimal two‐step selection process. (b) The selection efficiency of primary cells was evaluated by monitoring the expression level of the neuroblastoma marker GD2 using flow cytometry. (c) Cell viability of primary neuroblastoma cells was assessed after 24‐h treatments with various drug combinations, including cyclophosphamide (CP) (1, 2, and 4 mg/mL), doxorubicin (DOX) (1, 2, and 4 μg/mL), and vincristine (VCR) (1, 2, and 8 μg/mL). The optimal drug combination, with a Combination Index (CI) value of 0.5, is highlighted with an orange arrow. (d) A comparison of cell viability between the single‐drug treatments and the optimal combination is provided. Data represent the mean ± SD from three independent experiments. A one‐way ANOVA was conducted to compare the cell viability of the combination treatment with each single‐drug treatment, with significant differences observed (**p < 0.05, ***p < 0.001).

To further assess the feasibility of the personalized BioNDP neuroblastoma drug screening system, we utilized TH‐MYCN mice as a preclinical model to replicate the conditions observed in patients with neuroblastoma. Initially, we established a two‐step purification method for isolating primary neuroblastoma cells. This method consistently yielded purified cells exhibiting the morphological characteristics of adrenergic neuroblastoma cell types (Figure 2a), with high expression levels of GD2, a surface marker specific to neuroblastoma, as confirmed by FACS analysis. The percentage of GD2‐positive cells in both isolated primary tumor cells (97.6%) and SK‐N‐DZ cells (98.4%) was significantly higher than in HEK‐293 cells (0.1%) (Figure 2b). These findings indicate that this two‐step selection method effectively isolates primary neuroblastoma cells with high purity.

Building on preliminary assays using SK‐N‐DZ cells as the drug target, we further explored effective doses for the combinational testing of CP, DOX, and VCR on primary neuroblastoma cells using the BioNDP platform. The IC_50_ values were determined to be 3.38 mg/mL for CP, 4.55 μg/mL for DOX, and 13.27 μg/mL for VCR. Concentrations near the IC_50_ of each drug were selected to assess combination effects, specifically 1, 2, and 4 mg/mL of CP; 1, 2, and 4 μg/mL of DOX; and 1, 2, and 8 μg/mL of VCR. The results demonstrated that the combination of 2 mg/mL CP, 4 μg/mL DOX, and 2 μg/mL VCR significantly reduced cell viability to 16.1%, showing higher cytotoxicity compared to single‐drug treatments or other combinations (Figure 2c,d). Moreover, the CI value for this combination was 0.5, indicating a synergistic cytotoxic effect in primary neuroblastoma cells.

### 3.3 Evaluating the in vivo efficacy of a refined drug combination using TH‐MYCN mice

**FIGURE 3:**
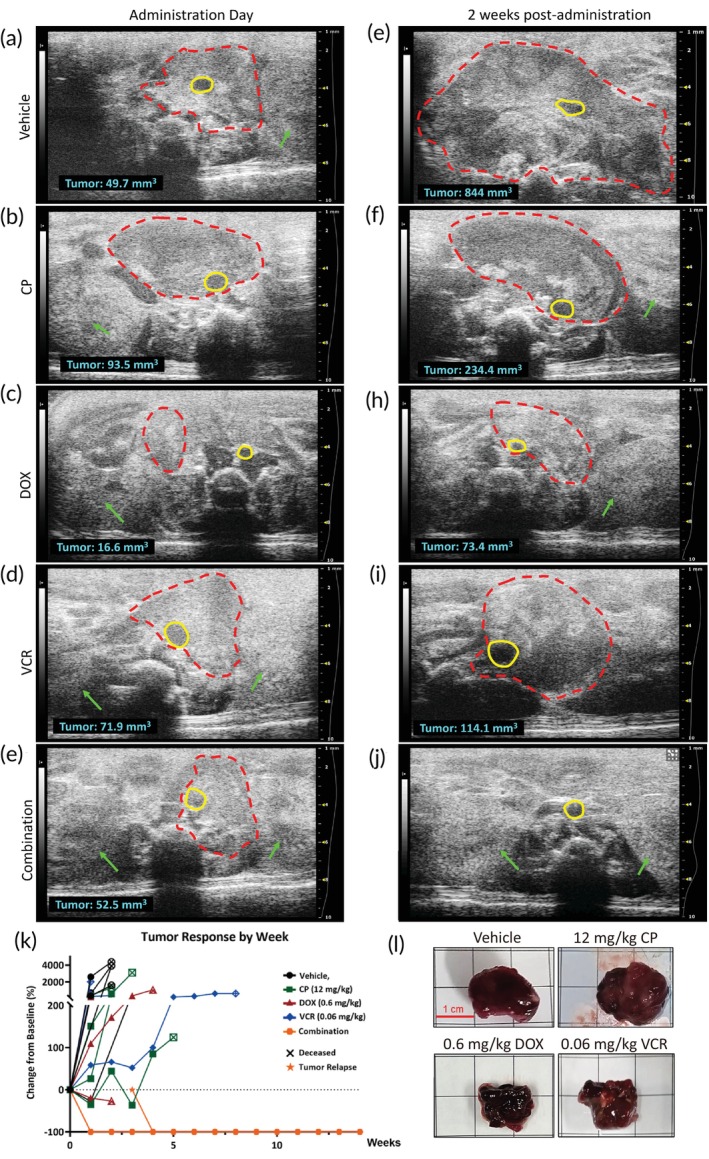
Sonograms depicting ventral views of tumors in TH‐MYCN mice. The TH‐MYCN mice were administered either a vehicle control, a single drug (12 mg/kg cyclophosphamide [CP], 0.6 mg/kg doxorubicin [DOX], 0.06 mg/kg vincristine [VCR]), or the selected combination therapy. The sonograms captured ventral views of the mice (a–e) before treatment and (f–i) after 2 weeks of treatment. (j) No tumor was detected in mice treated with the selected combination. Tumors are outlined with a red dotted line, while the dorsal aorta is marked by a yellow circle. The left and right kidneys are indicated by green arrows. A scale bar is displayed on the right side of each figure. (k) A spider plot illustrates the tumor response to each treatment, with changes in tumor volume evaluated by comparing the volume at each time point to the initial volume on the administration day (Day 0) (*n* = 5 for each group). Tumor volume was monitored one to two times per week using the VisualSonics VEVO‐2100 High‐Frequency Ultrasound system. (l) Representative images of tumors isolated from mice treated with the vehicle or a single dose of each drug on the experimental endpoint day. Tumors were collected on the day of sacrifice upon reaching the experimental endpoint and the size was measured on a 1‐cm grid paper.

**FIGURE 4:**
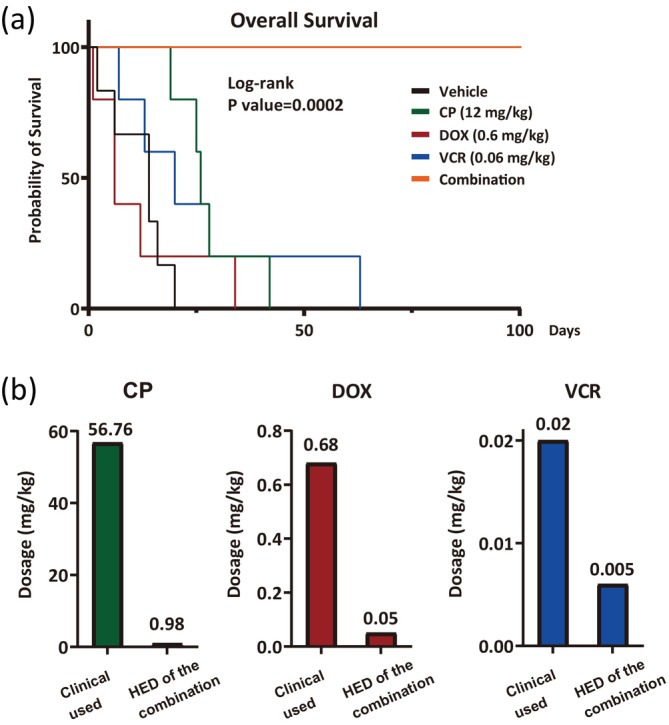
The refined drug combination treatment prolonged survival in TH‐MYCN mice by inhibiting tumor growth. TH‐MYCN mice were administered either a vehicle control, a single dose of each drug, or a combination of 12 mg/kg cyclophosphamide (CP), 0.6 mg/kg doxorubicin (DOX), and 0.06 mg/kg vincristine (VCR). (a) Survival curves for the TH‐MYCN mice under different treatments were generated using Kaplan–Meier survival analysis. The curves illustrate the survival of each TH‐MYCN mouse, with statistical analysis performed using the log‐rank test (Mantel‐Cox method) via GraphPad Prism 9.0.0 software (*n* = 5 for each group). (b) A comparison between the clinical dosage of each drug (CP, DOX, and VCR) and the human equivalent dose (HED) of the optimal combination identified in this study.

To assess the in vivo therapeutic efficacy of the drug combination identified through the BioNDP platform, we administered the therapy to TH‐MYCN mice. This administration protocol was adapted from the in vitro drug combination, following the algorithm developed in our previous study.^7^ Mice were systematically monitored using the VisualSonics VEVO‐2100 High‐Frequency Ultrasound system to detect tumor development, growth, or regression. Dosages of CP, DOX, and VCR for the animal experiments were determined based on the previously established algorithm^7^ and administered individually or in combination once tumors reached at least 0.5 cm in diameter (Figure 3a–e). The drug delivery procedure is illustrated in Figure S2. Following treatment, tumor sizes were monitored weekly using the ultrasound system. On average, tumor volumes in the vehicle‐treated mice increased more than 15‐fold within 2 weeks post‐administration (Figure 3f). In contrast, tumors in mice receiving single‐drug treatments continued to grow post‐administration (Figure 3g–i), increasing to over sixfold their original size within 2 weeks post‐administration (Figure 3k). Due to the rapid progression of tumors, several mice in the vehicle or single‐drug treatment groups required early euthanasia in accordance with animal care guidelines, resulting in the premature termination of the experiments (Figure 3k). In contrast, mice treated with the drug combination exhibited complete tumor regression within 2 weeks (Figure 3j,k). However, we observed tumor relapse in two mice during the fourth week post‐combination treatment, though re‐administration of the same combination led to sustained tumor regression until the study concluded (Figure 3k). At the endpoint of the experiment, significant tumor growth was observed in mice treated with the vehicle or single drugs, whereas those treated with the selected combination showed no detectable tumors (Figure 3l). Furthermore, survival analysis revealed that this combination therapy significantly extended the lifespan of TH‐MYCN mice compared to other treatments, in which mice either succumbed shortly after treatment or required euthanasia due to aggressive tumor growth (Figure 4a). To compare the dosages used in this study with clinically employed ones, we converted the dosages to human equivalent doses (HED) according to U.S. Food and Drug (FDA) guidelines.^42^ The conversion revealed that the HED of CP, DOX, and VCR in our study were approximately 1.72%, 7.35%, and 25% of the clinical doses, respectively (Figure 4b).

### 3.4 Assessment of side effects in TH‐MYCN mice treated with the refined drug combination

**FIGURE 5:**
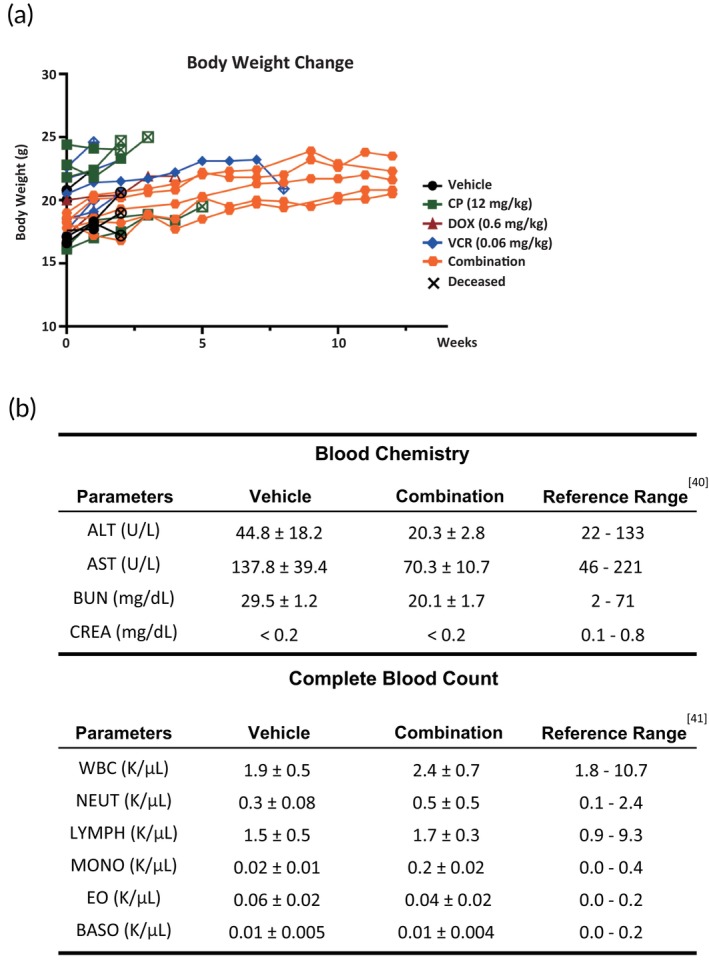
Evaluation of side effects in TH‐MYCN mice under different treatments. TH‐MYCN mice were randomly assigned to receive intraperitoneal injections of vehicle (0.9% saline) for 3 days, 12 mg/kg cyclophosphamide (CP) for 2 days, 0.6 mg/kg doxorubicin (DOX) for 3 days, 0.06 mg/kg vincristine (VCR) for 3 days, or a combination of these three drugs. (a) Body weight was monitored weekly for each mouse (*n* = 5 per group). (b) TH‐MYCN mice were treated with either vehicle (0.9% saline) for 3 days or the optimal drug combination (12 mg/kg CP for 2 days, 0.6 mg/kg DOX for 3 days, 0.06 mg/kg VCR for 3 days) and total blood was collected on the day after the final administration. Serum biochemical parameters and complete blood counts were analyzed by Bio‐Cando Incorporation (*n* = 4 per group).

Severe side effects are commonly associated with current treatment protocols for HR neuroblastoma, primarily due to the high doses of chemotherapeutic agents used in patients.^43^ The drug combination identified by the BioNDP platform significantly reduced the required doses of CP, DOX, and VCR, potentially mitigating the incidence of side effects. To verify this potential reduction, we evaluated the side effects of various treatments in TH‐MYCN mice, focusing on body weight changes and comprehensive blood analyses. Post‐administration, no significant changes in body weight were observed across all treatment groups (Figure 5a). Additionally, biochemical and hematological analyses of blood samples from mice treated with either the vehicle or combination therapy revealed that markers such as alanine aminotransferase (ALT), aspartate aminotransferase (AST), creatinine (CREA), and blood urea nitrogen (BUN)^44^, ^45^ remained within normal physiological ranges, indicating no significant liver or kidney toxicity (Figure 5b, top panel). Moreover, the cell counts for various blood cell types in mice treated with the combination therapy were comparable to those in the vehicle control group, suggesting that the selected drug combination maintained normal blood homeostasis in the murine models (Figure 5b, bottom panel).^46^, ^47^ On the other hand, liver, kidney, and spleen samples were collected, fixed, and stained with H&E. After reviewing the histological features of major organs (e.g., liver, kidney, and spleen), we did not find evidence of hepatotoxicity or renal toxicity in single‐agent therapies or combined therapy (Figure S6). These findings demonstrate that the drug combination identified by the BioNDP platform effectively inhibited tumor growth and induced tumor regression with minimal side effects.

Collectively, these results confirm the significant therapeutic efficacy of the selected drug combination, which successfully eradicated spontaneously developed neuroblastoma in TH‐MYCN mice. The administration of the selected combination at relatively low dosages suggests the potential of the BioNDP platform to identify novel drug dosages that minimize side effects, as corroborated by the blood and histological examinations. Overall, the in vivo results validate the therapeutic efficacy of the drug combination identified via the BioNDP platform, underscoring its potential to effectively treat neuroblastoma and improve overall survival outcomes in TH‐MYCN mice.

## 4 DISCUSSION

Traditional drug screening methods that utilize tumor cell lines in multi‐well plates have been criticized for their lack of reliability in generating clinically beneficial results, primarily due to the genomic heterogeneity within patient populations. Moreover, a previous study suggested that ideal drug profiling should involve 2000–6000 tumor cell lines encompassing at least 20 different tissue origins^48^—a requirement that significantly surpasses the capacity of existing screening platforms. Consequently, PDS, which employs patient‐derived primary cells, has emerged as a pivotal advancement in therapeutic strategies. While primary cells such as blood cells and dermal fibroblasts are frequently applied in PDS platforms,^49^ the acquisition of other primary cell types is often problematic, either due to their scarcity or limited availability, thereby posing significant challenges for compound screening. Conversely, existing PDS methods, including patient‐derived tumor organoids^50^ and patient‐derived xenografts,^51^ have shown the capability to replicate the complex characteristics of a tumor microenvironment. However, these approaches are hindered by limitations such as low throughput, high development costs, and the absence of a fully functional immune system.^52^, ^53^ In this study, we utilized our BioNDP platform as a PDS strategy to screen synergistic chemotherapeutic combinations specifically in primary HR neuroblastoma. The BioNDP platform is optimized for low cell numbers usage and multi‐drug screening, effectively addressing the limitations inherent in traditional screening platforms. We first validated the reliability of the BioNDP platform by comparing its performance to that of traditional 384‐well plates using the SK‐N‐DZ cell line. The results demonstrated comparable efficacy in cytotoxicity between the two platforms (Figure 1a). Additionally, while a prior study demonstrated that a PDMS chip could handle 16,000 primary cancer cells per single‐dose treatment,^54^ our approach significantly reduced the required cell number to just 100 cells per well, representing almost a 0.5% reduction compared to the previous study. Furthermore, our platform facilitates the assessment of synergistic effects from drug combinations, which is critical in identifying effective treatment strategies for HR neuroblastoma. The BioNDP platform enables rapid drug screening using patient‐derived samples, providing advantages such as lower costs and greater flexibility compared to traditional methods. These features collectively position the BioNDP platform as an ideal tool for initiating PDS, overcoming the existing limitations of traditional methods while significantly enhancing both efficiency and scalability. The platform's capacity for efficient and reliable PDS highlights its potential as a transformative tool in oncology.

HR neuroblastoma accounts for over 50% of newly diagnosed neuroblastoma cases. Intensive multi‐agent chemotherapy is typically employed during the induction phase to reduce tumor burden in patients with HR neuroblastoma. Although higher doses of chemotherapy may offer the potential to cure HR neuroblastoma, most pediatric patients are unable to complete the treatment due to severe side effects. Consequently, there is an urgent need for more effective and tolerable medications. To address this challenge, a more advanced drug screening platform is essential. In this study, we screened a drug combination of CP, DOX, and VCR using the BioNDP platform, which has previously demonstrated efficacy in drug screening across multiple cancer types and has been validated by in vivo animal models.^7^ We successfully identified the combination of CP, DOX, and VCR (CDV) and determined its cytotoxic efficacy using a neuroblastoma cell line (Figure 1b–d) and primary cells isolated from TH‐MYCN mice (Figure 2). Furthermore, this selected combination significantly reduced tumor volumes (Figure 3) and prolonged survival in the TH‐MYCN mouse model (Figure 4a). These in vivo results demonstrated complete tumor eradication over 12 weeks, in stark contrast to the outcomes from single‐drug or vehicle treatments, which resulted in continued tumor growth or mortality.

The Children's Oncology Group (COG) and the International Society of Pediatric Oncology Europe Neuroblastoma Group (SIOPEN) have developed various treatment regimens for HR neuroblastoma (Figure S5). In the current COG protocol, the CDV regimen includes CP at 70 mg/kg, DOX at approximately 0.68 mg/kg, and VCR at 0.022 mg/kg.^43^ However, when these clinical dosages were converted to their equivalents for use in mice based on the formula from a previous study,^42^ the dosage for CP reached 861 mg/kg, significantly exceeding the MTD for mice. This discrepancy underscores the unsuitability of directly applying standard clinical protocols in murine studies. Additionally, the administration of CP, DOX, and VCR has been associated with liver toxicity, as evidenced by elevated levels of ALT and AST.^44^ Moreover, treatment with CP at 50 mg/kg over 3 days led to a significant increase in CREA levels, indicating renal injury.^55^ These findings raise concerns about the potential for severe liver and kidney damage in children treated with the current COG protocol. In contrast, the HED of the CDV combination identified through the BioNDP platform were 0.98 mg/kg for CP, 0.05 mg/kg for DOX, and 0.005 mg/kg for VCR (Figure 4b), substantially lower than those used in clinical practice.^43^, ^56^, ^57^, ^58^, ^59^, ^60^, ^61^, ^62^, ^63^ Notably, mice treated with this combination maintained stable body weights, and comprehensive analyses of serum chemistry and blood counts revealed no significant hematological or non‐hematological adverse effects throughout the study period (Figure 5a,b). Furthermore, the histological examinations demonstrated normal liver parenchyma and renal glomeruli and tubules in different groups of mice (Figure S6), along with our biochemical data on liver and renal functions. Collectively, pathological evaluations and biochemical tests have provided critical biosafety data for our mice model. These findings provide strong evidence to support the safety of the drug combination identified through the BioNDP platform. Previous studies have integrated multiple therapies, including radiopharmaceutical iodine‐131 meta‐iodobenzylguanidine,^62^ anaplastic lymphoma kinase (ALK) inhibitors,^64^ autologous stem cell rescue,^57^ and anti‐GD2 monoclonal antibodies^65^—into the intensive induction regimen of the CDV combination. Unfortunately, these regimens frequently resulted in Grades 3–4 hematological and non‐hematological adverse effects during the clinical induction phase (Table 1). These observations highlight the critical requirements to reduce dosages during the induction phase while preserving therapeutic efficacy. Remarkably, the dosages administered in our study were 2‐ to 40‐fold lower than those reported in earlier studies. Moreover, this treatment regimen did not produce significant side effects, as indicated by stable body weights, normal levels of serum markers and blood cell counts, and unremarkable pathological features of organs. In recent years, immunotherapies—particularly GD2‐targeted therapies—have become increasingly central in the treatment of HR neuroblastoma. These therapies have been shown to enhance the effectiveness of chemotherapy when combined with other chemotherapeutic agents.^66^, ^67^ Additionally, PDMS‐based chips have been employed to develop tumor‐on‐a‐chip systems for screening combinations of chemotherapies and immunotherapies.^68^ Our current study, combined with these advancements, suggests that the BioNDP platform could be instrumental in developing innovative screening methods for combining chemotherapy and immunotherapy in the treatment of HR neuroblastoma.

**TABLE 1:** Comparison of the human equivalent dose (HED) of the combination in this study with common dosages used in high‐risk neuroblastoma patients.

Cyclophosphamide (mg/kg)	Doxorubicin (mg/kg)	Vincristine (mg/kg)	Regimen adjustment	Hematologic and non‐hematologic adverse effects	Refs.
0.98 × 2 days (HED)	0.05 × 3 days (HED)	0.005 × 3 days (HED)	• Low dosages identified from the BioNDP platform	• No obviously observed	This study
70 × 2 days	2.0^a^ × 3 days	0.067 × 3 days	• Reducing chemotherapy from seven to five cycles. • Immunotherapy with the anti‐GD2 3F8 monoclonal antibody	• Severe neutropenia • Mucositis, hearing deficits	50
27.03^a^ × 2 days	1.62 mg/kg^a^ × 3 days	0.04^a^ × 3 days	• The first attempt by Pediatric Oncology Group (POG) to intensive chemotherapy in combination with autologous stem cell rescue (ASCR)	• Grades 3 or 4 toxicities due to induction	51
40.54^a^ × 2 days	0.68^a^ × 3 days	0.01^a^ × 3 days	• Short topotecan‐based induction regimen	• Febrile neutropenia • Mucositis and diarrhea	52
56.76^a^ × 2 days	0.68^a^ × 3 days	0.02^a^ × 3 days			53
32.43^a^ × 3 days	0.81^a^ × 1 day	0.04^a^ × 3 days	• Delayed intensification chemotherapy	• Grades 3 to 4 neutropenia, thrombocytopenia	54
70 × 2 days	0.68^a^ × 3 days	0.022 mg/kg × 3 days	• Memorial Sloan Kettering Cancer Center (MSKCC)‐N5 regimen	• Grades 3 to 4 hematologic • Infections, gastrointestinal side effects (stomatitis, nausea, vomiting, and diarrhea)	37
70 × 2 days	0.68^a^ × 3 days	0.02^a^ × 3 days			55
54.05^a^ × 1 day	0.81^a^ × 2 days	0.04^a^ × 5 days	• Continual treatment of tumor cells with 131‐I‐MIBG radio‐chemotherapy for 1 month	• Grade 4 neutropenia • Grade 1 vomiting and mucositis	56
32.4^a^ × 1 day	NA	0.04^a^ × 1 day	• Delayed local treatment	• Grade 4 hematological adverse effects • Grade 4 non‐hematological adverse effects	57

^a^ Dosages translated from mg/m^2^ to mg/kg according to the FDA guideline.^36^

Abbreviation: BioNDP, Bioinspired Nanodroplet Processing.

More than half of the patients diagnosed with HR neuroblastoma experienced relapse despite undergoing comprehensive multimodal treatment, with the majority of recurrences occurring within the first 2 years post‐diagnosis.^69^ Previous studies have identified MYCN amplification as a key factor strongly associated with tumor relapse and aggressive growth.^70^ In our current study, we observed a similar pattern: two mice experienced tumor relapse following the administration of the selected drug combination. Initially, tumor volumes completely regressed within 2 weeks of treatment (Figure 3k). However, relapse was observed in two out of the five treated mice 4 weeks later (Figure 3k, indicated by a star). We hypothesize that the selected combination successfully eradicated the tumor in three mice, but it is likely that “dormant” cancer cells persisted in the remaining two mice due to ongoing MYCN expression, allowing these cells to evade the treatment and cause the observed relapses (Figure 3k). Notably, when the same combination was administered again, the relapsed tumors regressed completely, with no further relapses observed until the end of the study. We further hypothesize that the low drug dosages used in our study prevented the development of drug resistance while simultaneously producing synergistic therapeutic effects. Although previous research suggests that CP may indirectly inhibit MYCN expression,^71^ the precise mechanisms underlying the synergistic interactions among CP, DOX, and VCR, and their effects on MYCN, remain unclear and warrant further investigation.

## 5 CONCLUSION

In our study, we utilized the BioNDP platform to screen drug combinations specifically targeting HR neuroblastoma, a cancer known for its poor prognosis and severe treatment‐related side effects. This platform, which requires only a minimal number of cells for screening, demonstrated its effectiveness in the context of personalized medicine. Initially, we validated its efficacy using SK‐N‐DZ neuroblastoma cells. Subsequently, we identified a synergistic drug combination in primary tumor cells that significantly reduced tumor volumes in TH‐MYCN transgenic mice. Remarkably, the combination treatment completely eradicated tumors in the mice over 12 weeks. Throughout the treatment, the mice maintained stable body weights, with serum markers and blood cell counts remaining within normal ranges and unremarkable pathological features of the organs. These findings underscore the BioNDP platform's potential to enhance neuroblastoma treatment outcomes by identifying effective, low‐dose therapeutic combinations that minimize adverse effects.

## AUTHOR CONTRIBUTIONS

**Yen‐Tzu Liao:** Conceptualization; formal analysis; investigation; validation; visualization; writing – original draft; writing – review and editing. **Zhi‐Kai Yu:** Investigation; validation. **Yi‐Xun Huang:** Investigation; validation. **Kuan‐Hung Lin:** Writing – review and editing. **Ching‐Te Kuo:** Conceptualization; methodology; supervision; writing – review and editing. **Tsai‐Shan Yang:** Methodology. **Pei‐Yi Wu:** Methodology; resources; supervision; writing – review and editing. **Chi‐Tai Yeh:** Resources. **Yen‐Lin Liu:** Resources; methodology. **Chien‐Chin Chen:** Conceptualization; methodology; validation; writing – review and editing. **Chiung‐Nien Chen:** Conceptualization; funding acquisition; supervision. **Wen‐Ming Hsu:** Conceptualization; funding acquisition; supervision; writing – review and editing. **Hsinyu Lee:** Conceptualization; funding acquisition; project administration; supervision; writing – review and editing.

## CONFLICT OF INTEREST STATEMENT

The authors declare that they have no known competing financial interests or personal relationships that could have appeared to influence the work reported in this paper.

## Supporting information


**Figure S1.** Characteristics and procedure of the BioNDP screening platform. (A) Flow chart depicting the drug combination screening process on the BioNDP platform. (B) The reliability and precision of dispensing were demonstrated with a minimal coefficient of variation (CV) of 0.8%, achieved by dispensing 100 SK‐N‐DZ cells per well. Data analysis was performed using Imaging Lab Software (Bio‐Rad).

**Figure S2.** Treatment schema.

**Figure S3.** Assessment of SK‐N‐DZ cell line viability with CP and VCR using the BioNDP platform. The cell viability after treatment with (A) CP and (B) VCR was assessed using the CellTiter‐Glo® luminescent cell viability assay, with the IC_50_ values for each drug indicated. Data are presented as mean ± SD from three independent experiments.

**Figure S4.** Induction regimens of COG and SIOPEN in HR neuroblastoma clinical trials.

**Figure S5.** Assessment of cell viability in isolated primary neuroblastoma cells treated with CP, DOX, and VCR using the BioNDP platform. The IC_50_ values for each drug were determined. Data are presented as mean ± SD from three independent experiments.

**Figure S6.** Histological comparison of liver, kidney, and spleen tissues in control and treated groups.

## Data Availability

Data sharing not applicable to this article as no datasets were generated or analysed during the current study.
