# Supplementary figures and images for: Synergistic drug combination screening using a nanodroplet processing platform to enhance neuroblastoma treatment in TH‐MYCN transgenic mice
Source: Bioeng Transl Med. 2025 Mar 3;10(4):e70007. doi: 10.1002/btm2.70007 (PMC12284426; doi:10.1002/btm2.70007)

**(A)**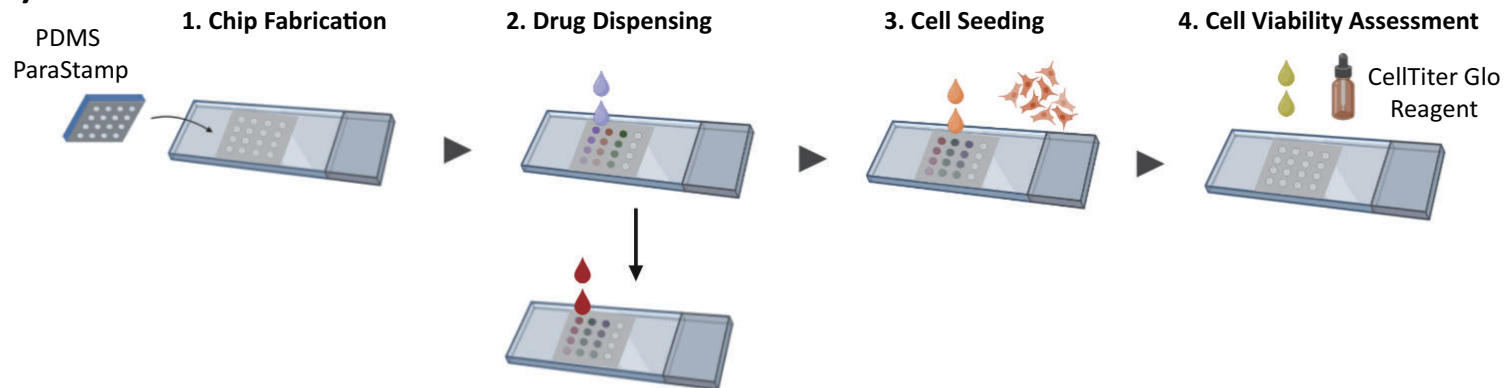**(B)**

Luminescence signal

Gray color

Pseudo color

100 cells/well (200 nL)

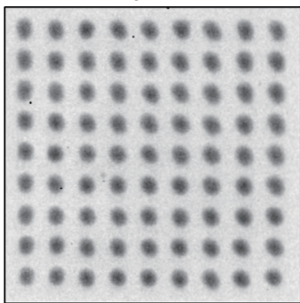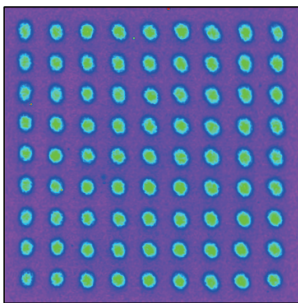

| Average RLU | STDEV   | CV% |
|-------------|---------|-----|
| 514,676.22  | 4217.97 | 0.8 |

Supplement: Supplementary file 1 — Figure S1. Characteristics and procedure of the BioNDP screening platform. (A) Flow chart depicting the drug combination screening process on the BioNDP platform. (B) The reliability and precision of dispensing were demonstrated with a minimal coefficient of variation (CV) of 0.8%, achieved by dispensing 100 SK‐N‐DZ cells per well. Data analysis was performed using Imaging Lab Software (Bio‐Rad). [file BTM2-10-e70007-s004.pdf]

### Cyclophosphamide

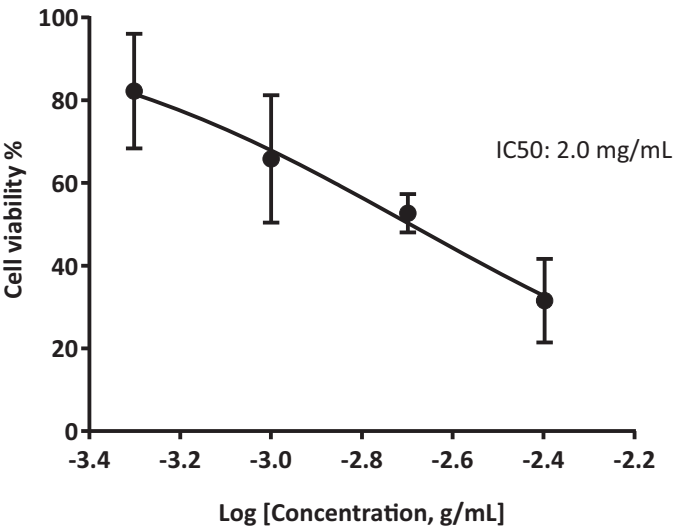

### Vincristine

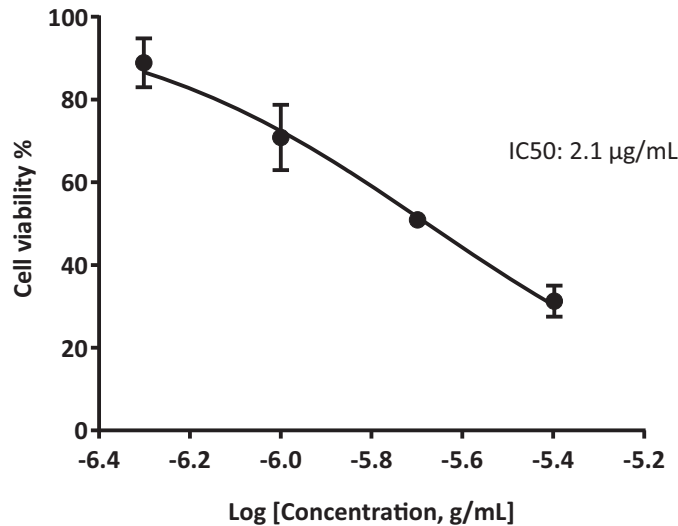

Supplement: Supplementary file 3 — Figure S3. Assessment of SK‐N‐DZ cell line viability with CP and VCR using the BioNDP platform. The cell viability after treatment with (A) CP and (B) VCR was assessed using the CellTiter‐Glo® luminescent cell viability assay, with the IC50 values for each drug indicated. Data are presented as mean ± SD from three independent experiments. [file BTM2-10-e70007-s005.pdf]

### Cyclophosphamide

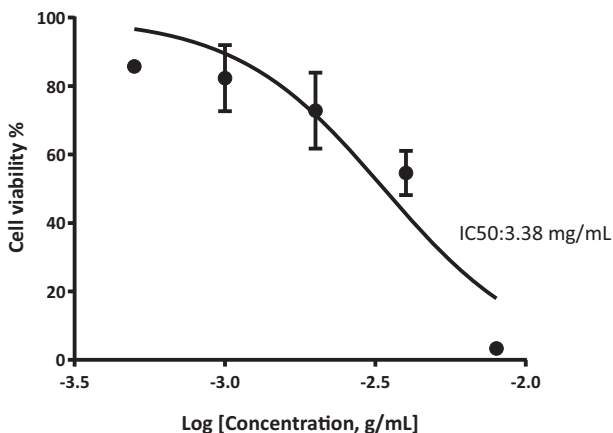

### Doxorubicin

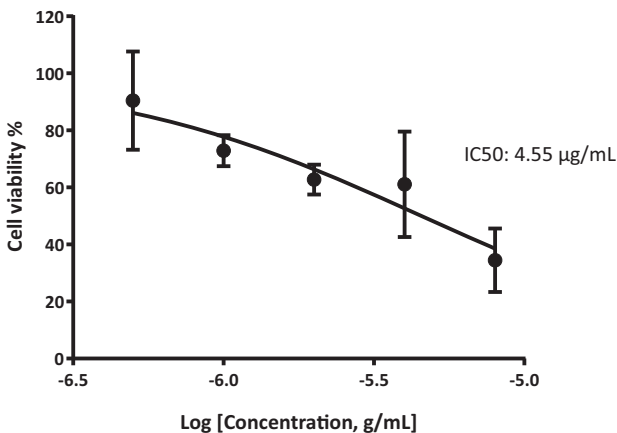

### Vincristine

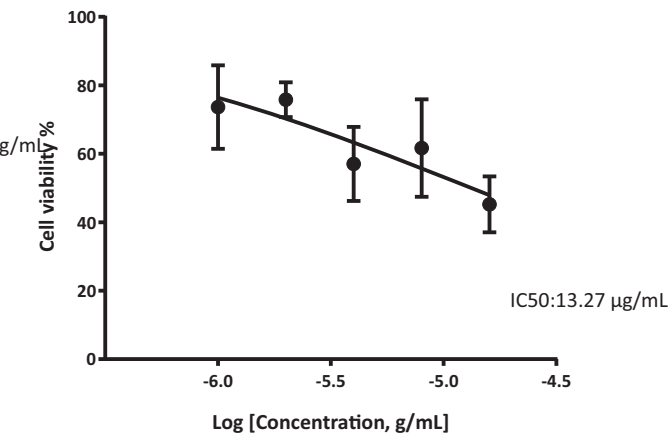

Supplement: Supplementary file 5 — Figure S5. Assessment of cell viability in isolated primary neuroblastoma cells treated with CP, DOX, and VCR using the BioNDP platform. The IC50 values for each drug were determined. Data are presented as mean ± SD from three independent experiments. [file BTM2-10-e70007-s001.pdf]

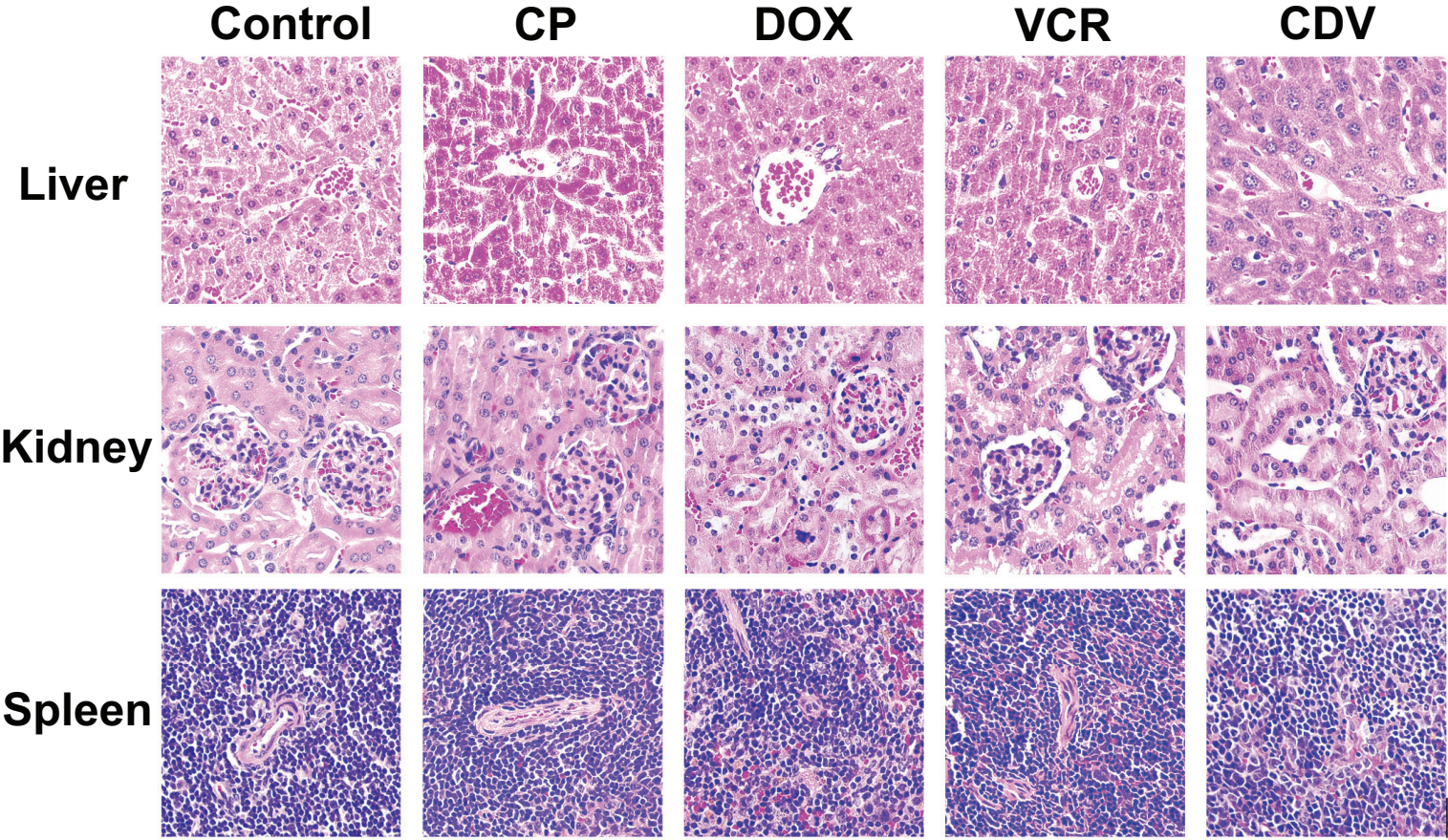

Supplement: Supplementary file 6 — Figure S6. Histological comparison of liver, kidney, and spleen tissues in control and treated groups. [file BTM2-10-e70007-s002.pdf]
