# Supplementary material for: Synergistic drug combination screening using a nanodroplet processing platform to enhance neuroblastoma treatment in TH‐MYCN transgenic mice
Source: Bioeng Transl Med. 2025 Mar 3;10(4):e70007. doi: 10.1002/btm2.70007 (PMC12284426; doi:10.1002/btm2.70007)
Supplement: Supplementary file 2 — Figure S2. Treatment schema. [file BTM2-10-e70007-s003.pdf]

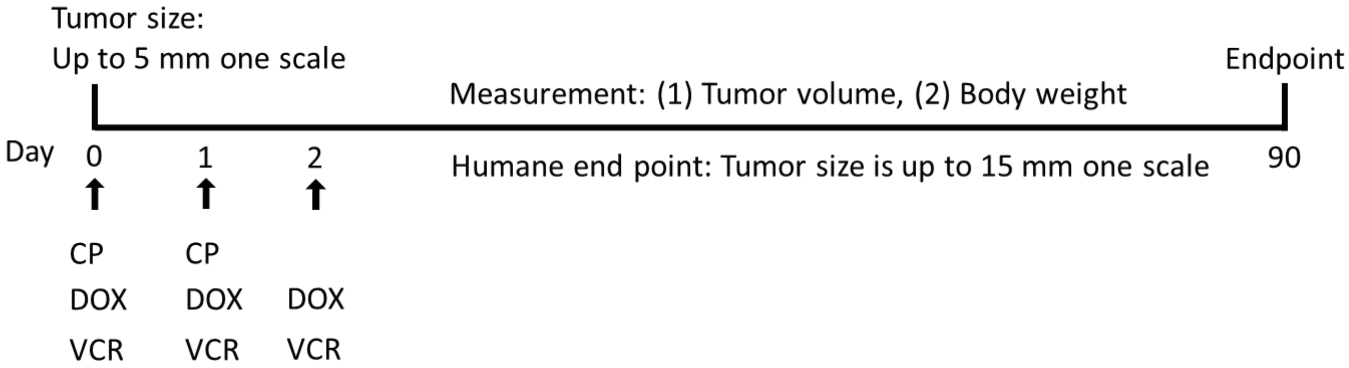
Tumor size:
Up to 5 mm one scale
Endpoint
Measurement: (1) Tumor volume, (2) Body weight
Day
0
1
2
90
Humane end point: Tumor size is up to 15 mm one scale
CP
CP
DOX
DOX
DOX
VCR
VCR
VCR
