# Supplementary material for: Synergistic drug combination screening using a nanodroplet processing platform to enhance neuroblastoma treatment in TH‐MYCN transgenic mice
Source: Bioeng Transl Med. 2025 Mar 3;10(4):e70007. doi: 10.1002/btm2.70007 (PMC12284426; doi:10.1002/btm2.70007)
Supplement: Supplementary file 4 — Figure S4. Induction regimens of COG and SIOPEN in HR neuroblastoma clinical trials. [file BTM2-10-e70007-s006.pdf]

SIOPEN used Rapid COJEC

Cycle	1	2	3	4	5	6	7	8
	A	B	C	B	A	B	C	B

**Course A**	Vincristine	1.5 mg/m^2 x 1 day
	Carboplatin	750 mg/m^2 x 1 day
	Etoposide	175 mg/m^2 x 2 days
**Course B**	Vincristine	1.5 mg/m^2 x 1 day
	Cisplatin	80 mg/m^2 x 1 day
**Course C**	Vincristine	1.5 mg/m^2 x 1 day
	Etoposide	175 mg/m^2 x 2 days
	Cyclophosphamide	1050 mg/m^2 x 2 days

COG used Modified N7

Cycle	1	2	3	4	5
	CDV	CDV	P/E	CDV	P/E

**Course CDV**	Cyclophosphamide	70 mg/kg x 2 day
	Doxorubicin	25 mg/m^2 x 3 day
	Vincristine	0.022 mg/kg x 3 days
**Course P/E**	Cisplatin	50 mg/m^2 x 1 day
	Etoposide	200 mg/m^2 x 1 day
